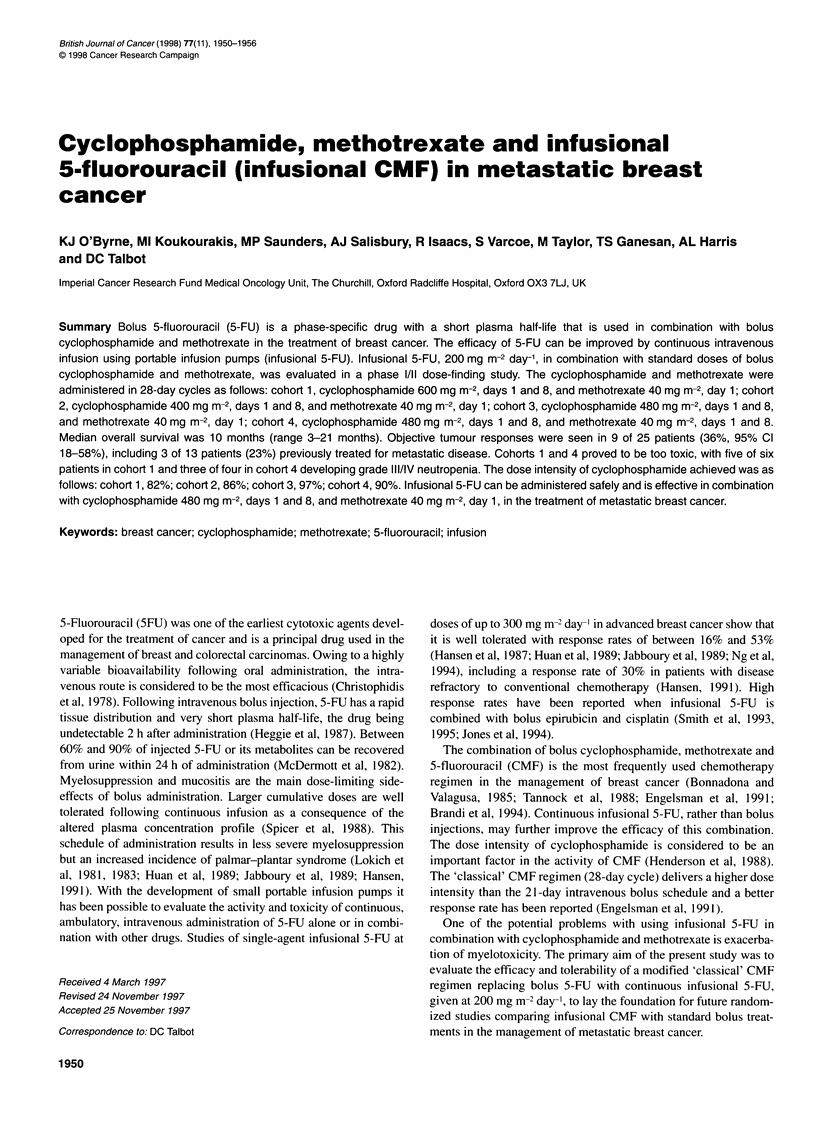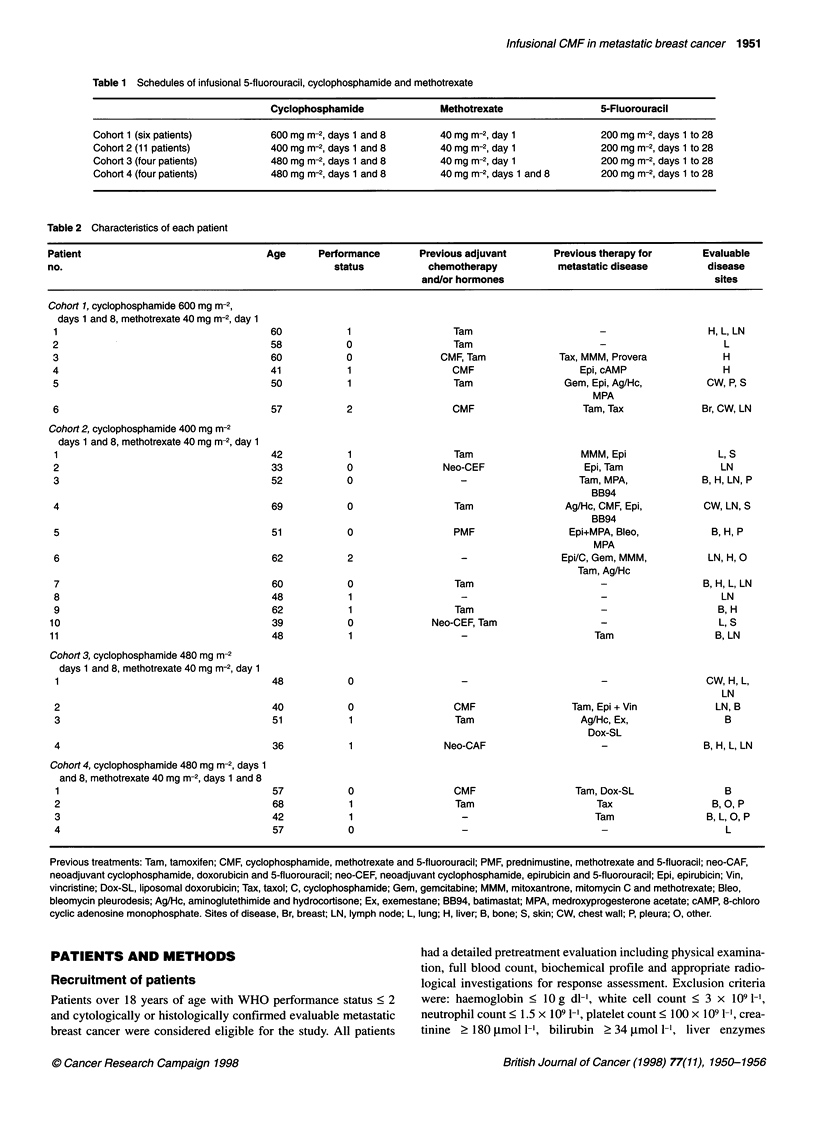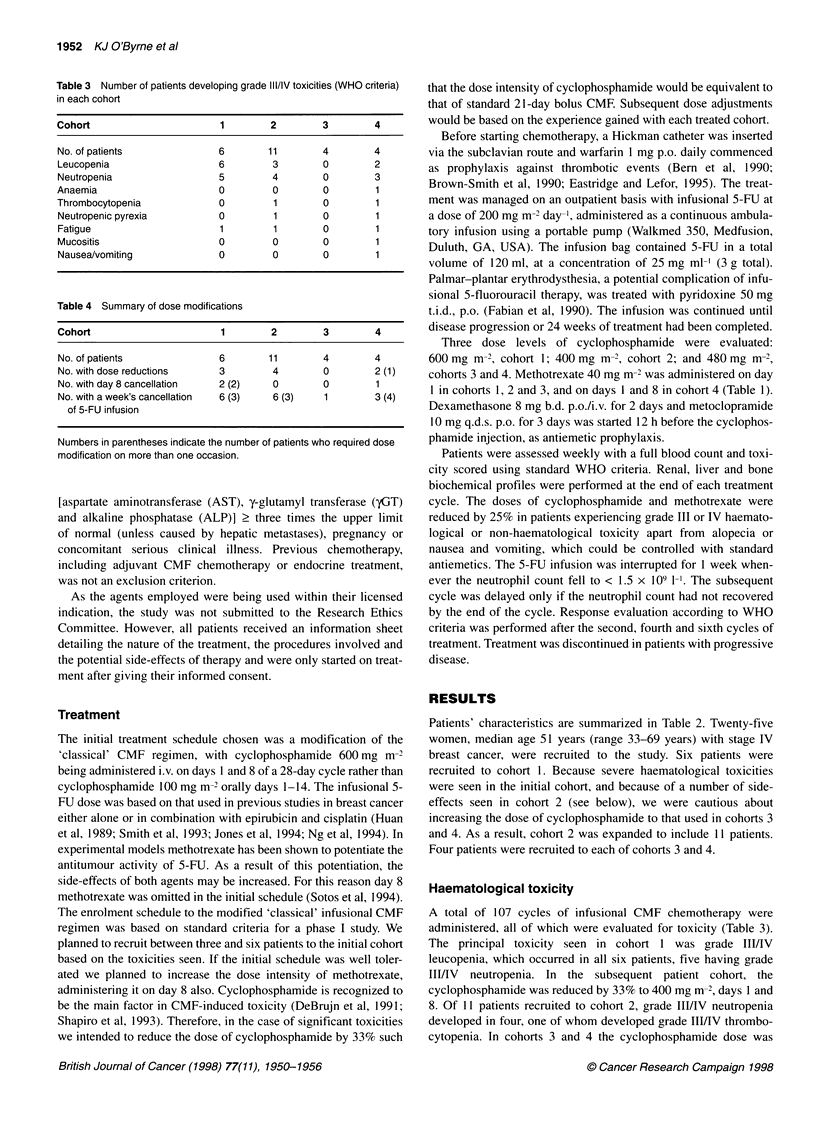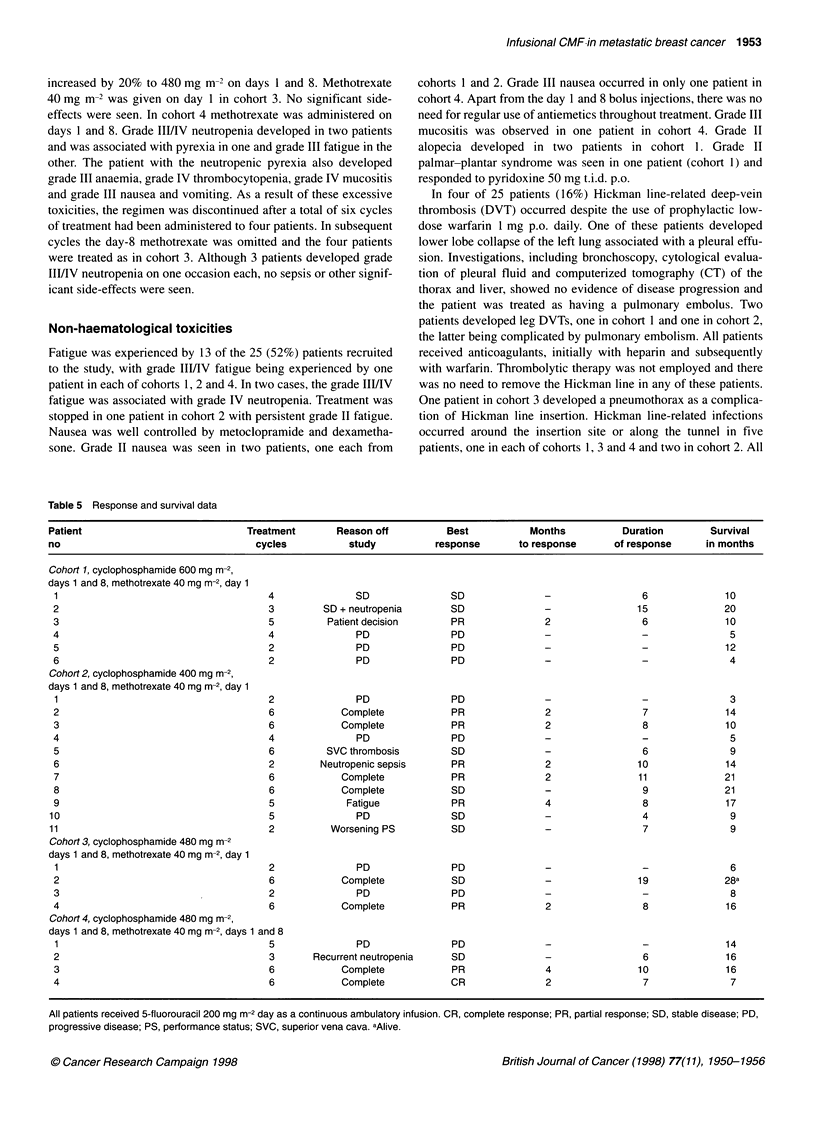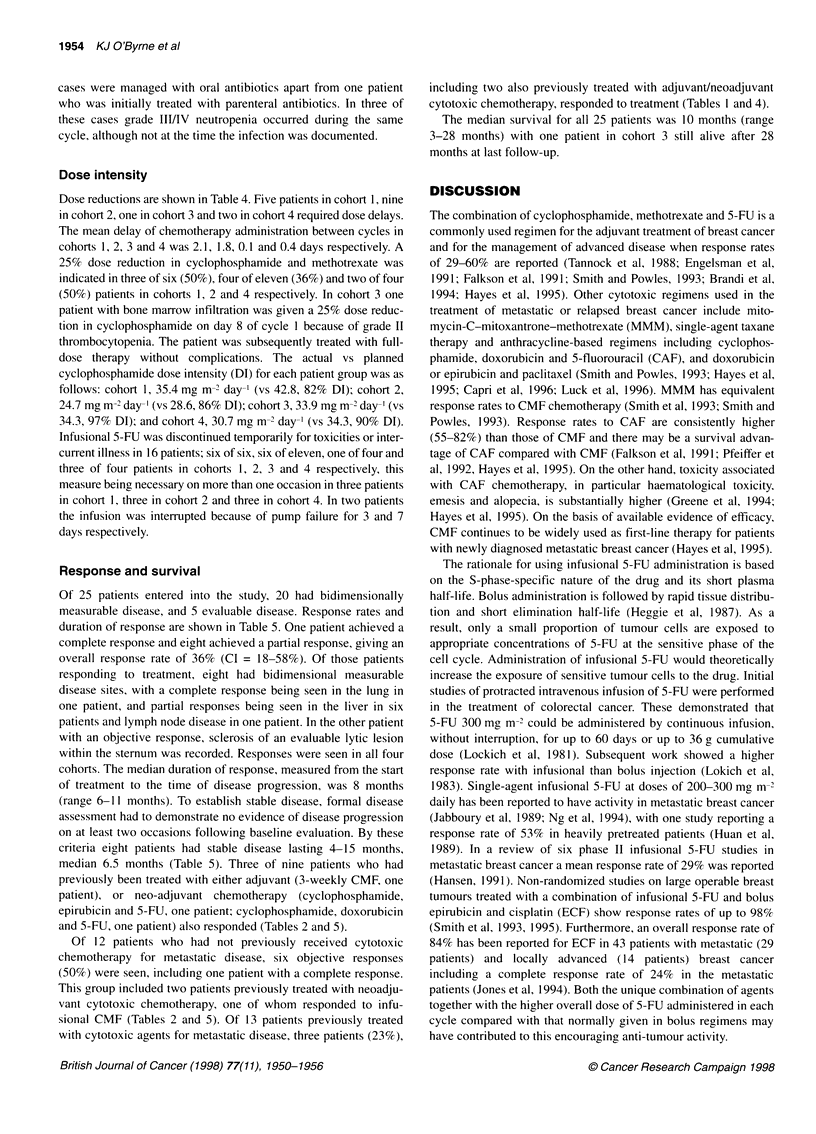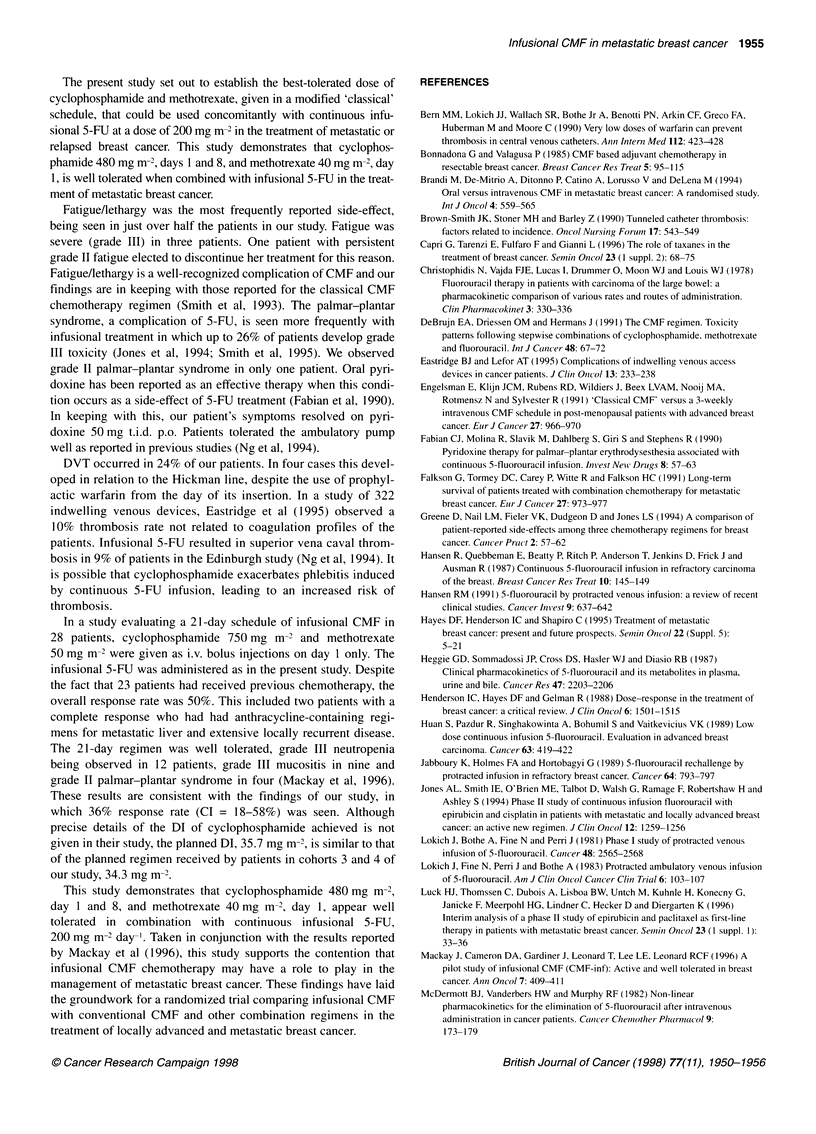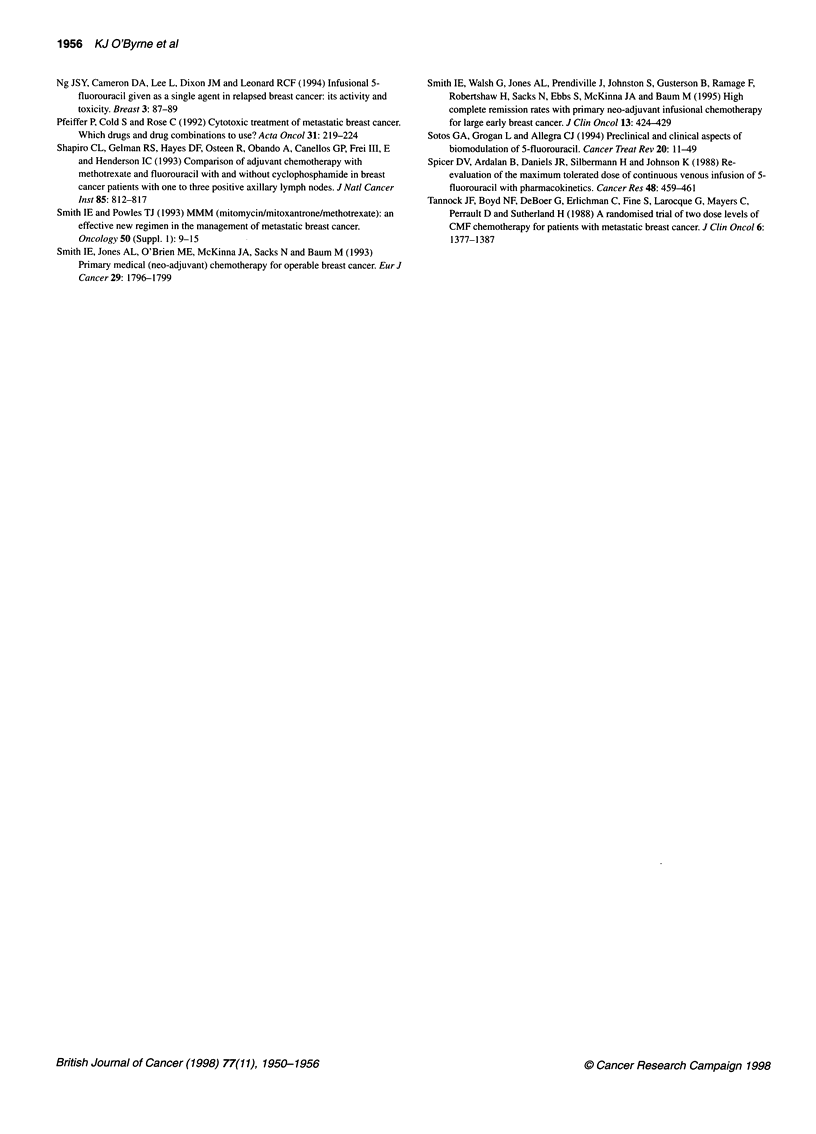# Cyclophosphamide, methotrexate and infusional 5-fluorouracil (infusional CMF) in metastatic breast cancer.

**DOI:** 10.1038/bjc.1998.323

**Published:** 1998-06

**Authors:** K. J. O'Byrne, M. I. Koukourakis, M. P. Saunders, A. J. Salisbury, R. Isaacs, S. Varcoe, M. Taylor, T. S. Ganesan, A. L. Harris, D. C. Talbot

**Affiliations:** Imperial Cancer Research Fund Medical Oncology Unit, The Churchill, Oxford Radcliffe Hospital, UK.

## Abstract

Bolus 5-fluorouracil (5-FU) is a phase-specific drug with a short plasma half-life that is used in combination with bolus cyclophosphamide and methotrexate in the treatment of breast cancer. The efficacy of 5-FU can be improved by continuous intravenous infusion using portable infusion pumps (infusional 5-FU). Infusional 5-FU, 200 mg m(-2) day(-1), in combination with standard doses of bolus cyclophosphamide and methotrexate, was evaluated in a phase I/II dose-finding study. The cyclophosphamide and methotrexate were administered in 28-day cycles as follows: cohort 1, cyclophosphamide 600 mg m(-2), days 1 and 8, and methotrexate 40 mg m(-2), day 1; cohort 2, cyclophosphamide 400 mg m(-2), days 1 and 8, and methotrexate 40 mg m(-2), day 1; cohort 3, cyclophosphamide 480 mg (m-2), days 1 and 8, and methotrexate 40 mg m(-2), day 1; cohort 4, cyclophosphamide 480 mg m(-2), days 1 and 8, and methotrexate 40 mg m(-2), days 1 and 8. Median overall survival was 10 months (range 3-21 months). Objective tumour responses were seen in 9 of 25 patients (36%, 95% CI 18-58%), including 3 of 13 patients (23%) previously treated for metastatic disease. Cohorts 1 and 4 proved to be too toxic, with five of six patients in cohort 1 and three of four in cohort 4 developing grade III/IV neutropenia. The dose intensity of cyclophosphamide achieved was as follows: cohort 1, 82%; cohort 2, 86%; cohort 3, 97%; cohort 4, 90%. Infusional 5-FU can be administered safely and is effective in combination with cyclophosphamide 480 mg m(-2), days 1 and 8, and methotrexate 40 mg m(-2), day 1, in the treatment of metastatic breast cancer.


British Journal of Cancer (1998) 77(11), 1950–1956
© 1998 Cancer Research Campaign

### Cyclophosphamide, methotrexate and infusional 5-fluorouracil (infusional CMF) in metastatic breast cancer

**KJ O'Byrne, MI Koukourakis, MP Saunders, AJ Salisbury, R Isaacs, S Varcoe, M Taylor, TS Ganesan, AL Harris and DC Talbot**

Imperial Cancer Research Fund Medical Oncology Unit, The Churchill, Oxford Radcliffe Hospital, Oxford OX3 7LJ, UK

**Summary** Bolus 5-fluorouracil (5-FU) is a phase-specific drug with a short plasma half-life that is used in combination with bolus cyclophosphamide and methotrexate in the treatment of breast cancer. The efficacy of 5-FU can be improved by continuous intravenous infusion using portable infusion pumps (infusional 5-FU). Infusional 5-FU, 200 mg $m^{-2}$ $day^{-1}$, in combination with standard doses of bolus cyclophosphamide and methotrexate, was evaluated in a phase I/II dose-finding study. The cyclophosphamide and methotrexate were administered in 28-day cycles as follows: cohort 1, cyclophosphamide 600 mg $m^{-2}$, days 1 and 8, and methotrexate 40 mg $m^{-2}$, day 1; cohort 2, cyclophosphamide 400 mg $m^{-2}$, days 1 and 8, and methotrexate 40 mg $m^{-2}$, day 1; cohort 3, cyclophosphamide 480 mg $m^{-2}$, days 1 and 8, and methotrexate 40 mg $m^{-2}$, day 1; cohort 4, cyclophosphamide 480 mg $m^{-2}$, days 1 and 8, and methotrexate 40 mg $m^{-2}$, days 1 and 8. Median overall survival was 10 months (range 3–21 months). Objective tumour responses were seen in 9 of 25 patients (36%, 95% CI 18–58%), including 3 of 13 patients (23%) previously treated for metastatic disease. Cohorts 1 and 4 proved to be too toxic, with five of six patients in cohort 1 and three of four in cohort 4 developing grade III/IV neutropenia. The dose intensity of cyclophosphamide achieved was as follows: cohort 1, 82%; cohort 2, 86%; cohort 3, 97%; cohort 4, 90%. Infusional 5-FU can be administered safely and is effective in combination with cyclophosphamide 480 mg $m^{-2}$, days 1 and 8, and methotrexate 40 mg $m^{-2}$, day 1, in the treatment of metastatic breast cancer.

**Keywords:** breast cancer; cyclophosphamide; methotrexate; 5-fluorouracil; infusion

5-Fluorouracil (5FU) was one of the earliest cytotoxic agents developed for the treatment of cancer and is a principal drug used in the management of breast and colorectal carcinomas. Owing to a highly variable bioavailability following oral administration, the intravenous route is considered to be the most efficacious (Christophidis et al, 1978). Following intravenous bolus injection, 5-FU has a rapid tissue distribution and very short plasma half-life, the drug being undetectable 2 h after administration (Heggie et al, 1987). Between 60% and 90% of injected 5-FU or its metabolites can be recovered from urine within 24 h of administration (McDermott et al, 1982). Myelosuppression and mucositis are the main dose-limiting side-effects of bolus administration. Larger cumulative doses are well tolerated following continuous infusion as a consequence of the altered plasma concentration profile (Spicer et al, 1988). This schedule of administration results in less severe myelosuppression but an increased incidence of palmar–plantar syndrome (Lokich et al, 1981, 1983; Huan et al, 1989; Jabboury et al, 1989; Hansen, 1991). With the development of small portable infusion pumps it has been possible to evaluate the activity and toxicity of continuous, ambulatory, intravenous administration of 5-FU alone or in combination with other drugs. Studies of single-agent infusional 5-FU at doses of up to 300 mg $m^{-2}$ $day^{-1}$ in advanced breast cancer show that it is well tolerated with response rates of between 16% and 53% (Hansen et al, 1987; Huan et al, 1989; Jabboury et al, 1989; Ng et al, 1994), including a response rate of 30% in patients with disease refractory to conventional chemotherapy (Hansen, 1991). High response rates have been reported when infusional 5-FU is combined with bolus epirubicin and cisplatin (Smith et al, 1993, 1995; Jones et al, 1994).

*Received 4 March 1997*
*Revised 24 November 1997*
*Accepted 25 November 1997*

*Correspondence to:* DC Talbot

The combination of bolus cyclophosphamide, methotrexate and 5-fluorouracil (CMF) is the most frequently used chemotherapy regimen in the management of breast cancer (Bonnadona and Valagusa, 1985; Tannock et al, 1988; Engelsman et al, 1991; Brandi et al, 1994). Continuous infusional 5-FU, rather than bolus injections, may further improve the efficacy of this combination. The dose intensity of cyclophosphamide is considered to be an important factor in the activity of CMF (Henderson et al, 1988). The 'classical' CMF regimen (28-day cycle) delivers a higher dose intensity than the 21-day intravenous bolus schedule and a better response rate has been reported (Engelsman et al, 1991).

One of the potential problems with using infusional 5-FU in combination with cyclophosphamide and methotrexate is exacerbation of myelotoxicity. The primary aim of the present study was to evaluate the efficacy and tolerability of a modified 'classical' CMF regimen replacing bolus 5-FU with continuous infusional 5-FU, given at 200 mg $m^{-2}$ $day^{-1}$, to lay the foundation for future randomized studies comparing infusional CMF with standard bolus treatments in the management of metastatic breast cancer.

**Table 1** Schedules of infusional 5-fluorouracil, cyclophosphamide and methotrexate

| | Cyclophosphamide | Methotrexate | 5-Fluorouracil |
|---|---|---|---|
| Cohort 1 (six patients) | 600 mg $m^{-2}$, days 1 and 8 | 40 mg $m^{-2}$, day 1 | 200 mg $m^{-2}$, days 1 to 28 |
| Cohort 2 (11 patients) | 400 mg $m^{-2}$, days 1 and 8 | 40 mg $m^{-2}$, day 1 | 200 mg $m^{-2}$, days 1 to 28 |
| Cohort 3 (four patients) | 480 mg $m^{-2}$, days 1 and 8 | 40 mg $m^{-2}$, day 1 | 200 mg $m^{-2}$, days 1 to 28 |
| Cohort 4 (four patients) | 480 mg $m^{-2}$, days 1 and 8 | 40 mg $m^{-2}$, days 1 and 8 | 200 mg $m^{-2}$, days 1 to 28 |

**Table 2** Characteristics of each patient

| Patient no. | Age | Performance status | Previous adjuvant chemotherapy and/or hormones | Previous therapy for metastatic disease | Evaluable disease sites |
|---|---|---|---|---|---|
| *Cohort 1,* cyclophosphamide 600 mg $m^{-2}$, days 1 and 8, methotrexate 40 mg $m^{-2}$, day 1 | | | | | |
| 1 | 60 | 1 | Tam | – | H, L, LN |
| 2 | 58 | 0 | Tam | – | L |
| 3 | 60 | 0 | CMF, Tam | Tax, MMM, Provera | H |
| 4 | 41 | 1 | CMF | Epi, cAMP | H |
| 5 | 50 | 1 | Tam | Gem, Epi, Ag/Hc, MPA | CW, P, S |
| 6 | 57 | 2 | CMF | Tam, Tax | Br, CW, LN |
| *Cohort 2,* cyclophosphamide 400 mg $m^{-2}$ days 1 and 8, methotrexate 40 mg $m^{-2}$, day 1 | | | | | |
| 1 | 42 | 1 | Tam | MMM, Epi | L, S |
| 2 | 33 | 0 | Neo-CEF | Epi, Tam | LN |
| 3 | 52 | 0 | – | Tam, MPA, BB94 | B, H, LN, P |
| 4 | 69 | 0 | Tam | Ag/Hc, CMF, Epi, BB94 | CW, LN, S |
| 5 | 51 | 0 | PMF | Epi+MPA, Bleo, MPA | B, H, P |
| 6 | 62 | 2 | – | Epi/C, Gem, MMM, Tam, Ag/Hc | LN, H, O |
| 7 | 60 | 0 | Tam | – | B, H, L, LN |
| 8 | 48 | 1 | – | – | LN |
| 9 | 62 | 1 | Tam | – | B, H |
| 10 | 39 | 0 | Neo-CEF, Tam | – | L, S |
| 11 | 48 | 1 | – | Tam | B, LN |
| *Cohort 3,* cyclophosphamide 480 mg $m^{-2}$ days 1 and 8, methotrexate 40 mg $m^{-2}$, day 1 | | | | | |
| 1 | 48 | 0 | – | – | CW, H, L, LN |
| 2 | 40 | 0 | CMF | Tam, Epi + Vin | LN, B |
| 3 | 51 | 1 | Tam | Ag/Hc, Ex, Dox-SL | B |
| 4 | 36 | 1 | Neo-CAF | – | B, H, L, LN |
| *Cohort 4,* cyclophosphamide 480 mg $m^{-2}$, days 1 and 8, methotrexate 40 mg $m^{-2}$, days 1 and 8 | | | | | |
| 1 | 57 | 0 | CMF | Tam, Dox-SL | B |
| 2 | 68 | 1 | Tam | Tax | B, O, P |
| 3 | 42 | 1 | – | Tam | B, L, O, P |
| 4 | 57 | 0 | – | – | L |

Previous treatments: Tam, tamoxifen; CMF, cyclophosphamide, methotrexate and 5-fluorouracil; PMF, prednimustine, methotrexate and 5-fluoracil; neo-CAF, neoadjuvant cyclophosphamide, doxorubicin and 5-fluorouracil; neo-CEF, neoadjuvant cyclophosphamide, epirubicin and 5-fluorouracil; Epi, epirubicin; Vin, vincristine; Dox-SL, liposomal doxorubicin; Tax, taxol; C, cyclophosphamide; Gem, gemcitabine; MMM, mitoxantrone, mitomycin C and methotrexate; Bleo, bleomycin pleurodesis; Ag/Hc, aminoglutethimide and hydrocortisone; Ex, exemestane; BB94, batimastat; MPA, medroxyprogesterone acetate; cAMP, 8-chloro cyclic adenosine monophosphate. Sites of disease, Br, breast; LN, lymph node; L, lung; H, liver; B, bone; S, skin; CW, chest wall; P, pleura; O, other.

### PATIENTS AND METHODS

### Recruitment of patients

Patients over 18 years of age with WHO performance status ≤ 2 and cytologically or histologically confirmed evaluable metastatic breast cancer were considered eligible for the study. All patients had a detailed pretreatment evaluation including physical examination, full blood count, biochemical profile and appropriate radiological investigations for response assessment. Exclusion criteria were: haemoglobin ≤ 10 g $dl^{-1}$, white cell count ≤ 3 × $10^9$ $l^{-1}$, neutrophil count ≤ 1.5 × $10^9$ $l^{-1}$, platelet count ≤ 100 × $10^9$ $l^{-1}$, creatinine ≥ 180 μmol $l^{-1}$, bilirubin ≥ 34 μmol $l^{-1}$, liver enzymes

© Cancer Research Campaign 1998 

Table 3 Number of patients developing grade III/IV toxicities (WHO criteria) in each cohort

| Cohort | 1 | 2 | 3 | 4 |
|---|---|---|---|---|
| No. of patients | 6 | 11 | 4 | 4 |
| Leucopenia | 6 | 3 | 0 | 2 |
| Neutropenia | 5 | 4 | 0 | 3 |
| Anaemia | 0 | 0 | 0 | 1 |
| Thrombocytopenia | 0 | 1 | 0 | 1 |
| Neutropenic pyrexia | 0 | 1 | 0 | 1 |
| Fatigue | 1 | 1 | 0 | 1 |
| Mucositis | 0 | 0 | 0 | 1 |
| Nausea/vomiting | 0 | 0 | 0 | 1 |

Table 4 Summary of dose modifications

| Cohort | 1 | 2 | 3 | 4 |
|---|---|---|---|---|
| No. of patients | 6 | 11 | 4 | 4 |
| No. with dose reductions | 3 | 4 | 0 | 2 (1) |
| No. with day 8 cancellation | 2 (2) | 0 | 0 | 1 |
| No. with a week's cancellation of 5-FU infusion | 6 (3) | 6 (3) | 1 | 3 (4) |

Numbers in parentheses indicate the number of patients who required dose modification on more than one occasion.

[aspartate aminotransferase (AST), γ-glutamyl transferase (γGT) and alkaline phosphatase (ALP)] ≥ three times the upper limit of normal (unless caused by hepatic metastases), pregnancy or concomitant serious clinical illness. Previous chemotherapy, including adjuvant CMF chemotherapy or endocrine treatment, was not an exclusion criterion.

As the agents employed were being used within their licensed indication, the study was not submitted to the Research Ethics Committee. However, all patients received an information sheet detailing the nature of the treatment, the procedures involved and the potential side-effects of therapy and were only started on treatment after giving their informed consent.

### Treatment

The initial treatment schedule chosen was a modification of the 'classical' CMF regimen, with cyclophosphamide 600 mg $m^{-2}$ being administered i.v. on days 1 and 8 of a 28-day cycle rather than cyclophosphamide 100 mg $m^{-2}$ orally days 1–14. The infusional 5-FU dose was based on that used in previous studies in breast cancer either alone or in combination with epirubicin and cisplatin (Huan et al, 1989; Smith et al, 1993; Jones et al, 1994; Ng et al, 1994). In experimental models methotrexate has been shown to potentiate the antitumour activity of 5-FU. As a result of this potentiation, the side-effects of both agents may be increased. For this reason day 8 methotrexate was omitted in the initial schedule (Sotos et al, 1994). The enrolment schedule to the modified 'classical' infusional CMF regimen was based on standard criteria for a phase I study. We planned to recruit between three and six patients to the initial cohort based on the toxicities seen. If the initial schedule was well tolerated we planned to increase the dose intensity of methotrexate, administering it on day 8 also. Cyclophosphamide is recognized to be the main factor in CMF-induced toxicity (DeBrujn et al, 1991; Shapiro et al, 1993). Therefore, in the case of significant toxicities we intended to reduce the dose of cyclophosphamide by 33% such that the dose intensity of cyclophosphamide would be equivalent to that of standard 21-day bolus CMF. Subsequent dose adjustments would be based on the experience gained with each treated cohort.

Before starting chemotherapy, a Hickman catheter was inserted via the subclavian route and warfarin 1 mg p.o. daily commenced as prophylaxis against thrombotic events (Bern et al, 1990; Brown-Smith et al, 1990; Eastridge and Lefor, 1995). The treatment was managed on an outpatient basis with infusional 5-FU at a dose of 200 mg $m^{-2}$ $day^{-1}$, administered as a continuous ambulatory infusion using a portable pump (Walkmed 350, Medfusion, Duluth, GA, USA). The infusion bag contained 5-FU in a total volume of 120 ml, at a concentration of 25 mg $ml^{-1}$ (3 g total). Palmar–plantar erythrodysthesia, a potential complication of infusional 5-fluorouracil therapy, was treated with pyridoxine 50 mg t.i.d., p.o. (Fabian et al, 1990). The infusion was continued until disease progression or 24 weeks of treatment had been completed.

Three dose levels of cyclophosphamide were evaluated: 600 mg $m^{-2}$, cohort 1; 400 mg $m^{-2}$, cohort 2; and 480 mg $m^{-2}$, cohorts 3 and 4. Methotrexate 40 mg $m^{-2}$ was administered on day 1 in cohorts 1, 2 and 3, and on days 1 and 8 in cohort 4 (Table 1). Dexamethasone 8 mg b.d. p.o./i.v. for 2 days and metoclopramide 10 mg q.d.s. p.o. for 3 days was started 12 h before the cyclophosphamide injection, as antiemetic prophylaxis.

Patients were assessed weekly with a full blood count and toxicity scored using standard WHO criteria. Renal, liver and bone biochemical profiles were performed at the end of each treatment cycle. The doses of cyclophosphamide and methotrexate were reduced by 25% in patients experiencing grade III or IV haematological or non-haematological toxicity apart from alopecia or nausea and vomiting, which could be controlled with standard antiemetics. The 5-FU infusion was interrupted for 1 week whenever the neutrophil count fell to $< 1.5 \times 10^9$ $l^{-1}$. The subsequent cycle was delayed only if the neutrophil count had not recovered by the end of the cycle. Response evaluation according to WHO criteria was performed after the second, fourth and sixth cycles of treatment. Treatment was discontinued in patients with progressive disease.

### RESULTS

Patients' characteristics are summarized in Table 2. Twenty-five women, median age 51 years (range 33–69 years) with stage IV breast cancer, were recruited to the study. Six patients were recruited to cohort 1. Because severe haematological toxicities were seen in the initial cohort, and because of a number of side-effects seen in cohort 2 (see below), we were cautious about increasing the dose of cyclophosphamide to that used in cohorts 3 and 4. As a result, cohort 2 was expanded to include 11 patients. Four patients were recruited to each of cohorts 3 and 4.

### Haematological toxicity

A total of 107 cycles of infusional CMF chemotherapy were administered, all of which were evaluated for toxicity (Table 3). The principal toxicity seen in cohort 1 was grade III/IV leucopenia, which occurred in all six patients, five having grade III/IV neutropenia. In the subsequent patient cohort, the cyclophosphamide was reduced by 33% to 400 mg $m^{-2}$, days 1 and 8. Of 11 patients recruited to cohort 2, grade III/IV neutropenia developed in four, one of whom developed grade III/IV thrombocytopenia. In cohorts 3 and 4 the cyclophosphamide dose was

 © Cancer Research Campaign 1998

increased by 20% to 480 mg m$^{-2}$ on days 1 and 8. Methotrexate 40 mg m$^{-2}$ was given on day 1 in cohort 3. No significant side-effects were seen. In cohort 4 methotrexate was administered on days 1 and 8. Grade III/IV neutropenia developed in two patients and was associated with pyrexia in one and grade III fatigue in the other. The patient with the neutropenic pyrexia also developed grade III anaemia, grade IV thrombocytopenia, grade IV mucositis and grade III nausea and vomiting. As a result of these excessive toxicities, the regimen was discontinued after a total of six cycles of treatment had been administered to four patients. In subsequent cycles the day-8 methotrexate was omitted and the four patients were treated as in cohort 3. Although 3 patients developed grade III/IV neutropenia on one occasion each, no sepsis or other significant side-effects were seen.

### Non-haematological toxicities

Fatigue was experienced by 13 of the 25 (52%) patients recruited to the study, with grade III/IV fatigue being experienced by one patient in each of cohorts 1, 2 and 4. In two cases, the grade III/IV fatigue was associated with grade IV neutropenia. Treatment was stopped in one patient in cohort 2 with persistent grade II fatigue. Nausea was well controlled by metoclopramide and dexamethasone. Grade II nausea was seen in two patients, one each from cohorts 1 and 2. Grade III nausea occurred in only one patient in cohort 4. Apart from the day 1 and 8 bolus injections, there was no need for regular use of antiemetics throughout treatment. Grade III mucositis was observed in one patient in cohort 4. Grade II alopecia developed in two patients in cohort 1. Grade II palmar–plantar syndrome was seen in one patient (cohort 1) and responded to pyridoxine 50 mg t.i.d. p.o.

In four of 25 patients (16%) Hickman line-related deep-vein thrombosis (DVT) occurred despite the use of prophylactic low-dose warfarin 1 mg p.o. daily. One of these patients developed lower lobe collapse of the left lung associated with a pleural effusion. Investigations, including bronchoscopy, cytological evaluation of pleural fluid and computerized tomography (CT) of the thorax and liver, showed no evidence of disease progression and the patient was treated as having a pulmonary embolus. Two patients developed leg DVTs, one in cohort 1 and one in cohort 2, the latter being complicated by pulmonary embolism. All patients received anticoagulants, initially with heparin and subsequently with warfarin. Thrombolytic therapy was not employed and there was no need to remove the Hickman line in any of these patients. One patient in cohort 3 developed a pneumothorax as a complication of Hickman line insertion. Hickman line-related infections occurred around the insertion site or along the tunnel in five patients, one in each of cohorts 1, 3 and 4 and two in cohort 2. All

**Table 5** Response and survival data

| Patient no | Treatment cycles | Reason off study | Best response | Months to response | Duration of response | Survival in months |
|---|---|---|---|---|---|---|
| *Cohort 1,* cyclophosphamide 600 mg m$^{-2}$, days 1 and 8, methotrexate 40 mg m$^{-2}$, day 1 | | | | | | |
| 1 | 4 | SD | SD | – | 6 | 10 |
| 2 | 3 | SD + neutropenia | SD | – | 15 | 20 |
| 3 | 5 | Patient decision | PR | 2 | 6 | 10 |
| 4 | 4 | PD | PD | – | – | 5 |
| 5 | 2 | PD | PD | – | – | 12 |
| 6 | 2 | PD | PD | – | – | 4 |
| *Cohort 2,* cyclophosphamide 400 mg m$^{-2}$, days 1 and 8, methotrexate 40 mg m$^{-2}$, day 1 | | | | | | |
| 1 | 2 | PD | PD | – | – | 3 |
| 2 | 6 | Complete | PR | 2 | 7 | 14 |
| 3 | 6 | Complete | PR | 2 | 8 | 10 |
| 4 | 4 | PD | PD | – | – | 5 |
| 5 | 6 | SVC thrombosis | SD | – | 6 | 9 |
| 6 | 2 | Neutropenic sepsis | PR | 2 | 10 | 14 |
| 7 | 6 | Complete | PR | 2 | 11 | 21 |
| 8 | 6 | Complete | SD | – | 9 | 21 |
| 9 | 5 | Fatigue | PR | 4 | 8 | 17 |
| 10 | 5 | PD | SD | – | 4 | 9 |
| 11 | 2 | Worsening PS | SD | – | 7 | 9 |
| *Cohort 3,* cyclophosphamide 480 mg m$^{-2}$ days 1 and 8, methotrexate 40 mg m$^{-2}$, day 1 | | | | | | |
| 1 | 2 | PD | PD | – | – | 6 |
| 2 | 6 | Complete | SD | – | 19 | 28[a] |
| 3 | 2 | PD | PD | – | – | 8 |
| 4 | 6 | Complete | PR | 2 | 8 | 16 |
| *Cohort 4,* cyclophosphamide 480 mg m$^{-2}$, days 1 and 8, methotrexate 40 mg m$^{-2}$, days 1 and 8 | | | | | | |
| 1 | 5 | PD | PD | – | – | 14 |
| 2 | 3 | Recurrent neutropenia | SD | – | 6 | 16 |
| 3 | 6 | Complete | PR | 4 | 10 | 16 |
| 4 | 6 | Complete | CR | 2 | 7 | 7 |

All patients received 5-fluorouracil 200 mg m$^{-2}$ day as a continuous ambulatory infusion. CR, complete response; PR, partial response; SD, stable disease; PD, progressive disease; PS, performance status; SVC, superior vena cava. [a]Alive.

© Cancer Research Campaign 1998

cases were managed with oral antibiotics apart from one patient who was initially treated with parenteral antibiotics. In three of these cases grade III/IV neutropenia occurred during the same cycle, although not at the time the infection was documented.

### Dose intensity

Dose reductions are shown in Table 4. Five patients in cohort 1, nine in cohort 2, one in cohort 3 and two in cohort 4 required dose delays. The mean delay of chemotherapy administration between cycles in cohorts 1, 2, 3 and 4 was 2.1, 1.8, 0.1 and 0.4 days respectively. A 25% dose reduction in cyclophosphamide and methotrexate was indicated in three of six (50%), four of eleven (36%) and two of four (50%) patients in cohorts 1, 2 and 4 respectively. In cohort 3 one patient with bone marrow infiltration was given a 25% dose reduction in cyclophosphamide on day 8 of cycle 1 because of grade II thrombocytopenia. The patient was subsequently treated with full-dose therapy without complications. The actual vs planned cyclophosphamide dose intensity (DI) for each patient group was as follows: cohort 1, 35.4 mg $m^{-2}$ $day^{-1}$ (vs 42.8, 82% DI); cohort 2, 24.7 mg $m^{-2}$ $day^{-1}$ (vs 28.6, 86% DI); cohort 3, 33.9 mg $m^{-2}$ $day^{-1}$ (vs 34.3, 97% DI); and cohort 4, 30.7 mg $m^{-2}$ $day^{-1}$ (vs 34.3, 90% DI). Infusional 5-FU was discontinued temporarily for toxicities or intercurrent illness in 16 patients; six of six, six of eleven, one of four and three of four patients in cohorts 1, 2, 3 and 4 respectively, this measure being necessary on more than one occasion in three patients in cohort 1, three in cohort 2 and three in cohort 4. In two patients the infusion was interrupted because of pump failure for 3 and 7 days respectively.

### Response and survival

Of 25 patients entered into the study, 20 had bidimensionally measurable disease, and 5 evaluable disease. Response rates and duration of response are shown in Table 5. One patient achieved a complete response and eight achieved a partial response, giving an overall response rate of 36% (CI = 18–58%). Of those patients responding to treatment, eight had bidimensional measurable disease sites, with a complete response being seen in the lung in one patient, and partial responses being seen in the liver in six patients and lymph node disease in one patient. In the other patient with an objective response, sclerosis of an evaluable lytic lesion within the sternum was recorded. Responses were seen in all four cohorts. The median duration of response, measured from the start of treatment to the time of disease progression, was 8 months (range 6–11 months). To establish stable disease, formal disease assessment had to demonstrate no evidence of disease progression on at least two occasions following baseline evaluation. By these criteria eight patients had stable disease lasting 4–15 months, median 6.5 months (Table 5). Three of nine patients who had previously been treated with either adjuvant (3-weekly CMF, one patient), or neo-adjuvant chemotherapy (cyclophosphamide, epirubicin and 5-FU, one patient; cyclophosphamide, doxorubicin and 5-FU, one patient) also responded (Tables 2 and 5).

Of 12 patients who had not previously received cytotoxic chemotherapy for metastatic disease, six objective responses (50%) were seen, including one patient with a complete response. This group included two patients previously treated with neoadjuvant cytotoxic chemotherapy, one of whom responded to infusional CMF (Tables 2 and 5). Of 13 patients previously treated with cytotoxic agents for metastatic disease, three patients (23%), including two also previously treated with adjuvant/neoadjuvant cytotoxic chemotherapy, responded to treatment (Tables 1 and 4).

The median survival for all 25 patients was 10 months (range 3–28 months) with one patient in cohort 3 still alive after 28 months at last follow-up.

### DISCUSSION

The combination of cyclophosphamide, methotrexate and 5-FU is a commonly used regimen for the adjuvant treatment of breast cancer and for the management of advanced disease when response rates of 29–60% are reported (Tannock et al, 1988; Engelsman et al, 1991; Falkson et al, 1991; Smith and Powles, 1993; Brandi et al, 1994; Hayes et al, 1995). Other cytotoxic regimens used in the treatment of metastatic or relapsed breast cancer include mitomycin-C–mitoxantrone–methotrexate (MMM), single-agent taxane therapy and anthracycline-based regimens including cyclophosphamide, doxorubicin and 5-fluorouracil (CAF), and doxorubicin or epirubicin and paclitaxel (Smith and Powles, 1993; Hayes et al, 1995; Capri et al, 1996; Luck et al, 1996). MMM has equivalent response rates to CMF chemotherapy (Smith et al, 1993; Smith and Powles, 1993). Response rates to CAF are consistently higher (55–82%) than those of CMF and there may be a survival advantage of CAF compared with CMF (Falkson et al, 1991; Pfeiffer et al, 1992, Hayes et al, 1995). On the other hand, toxicity associated with CAF chemotherapy, in particular haematological toxicity, emesis and alopecia, is substantially higher (Greene et al, 1994; Hayes et al, 1995). On the basis of available evidence of efficacy, CMF continues to be widely used as first-line therapy for patients with newly diagnosed metastatic breast cancer (Hayes et al, 1995).

The rationale for using infusional 5-FU administration is based on the S-phase-specific nature of the drug and its short plasma half-life. Bolus administration is followed by rapid tissue distribution and short elimination half-life (Heggie et al, 1987). As a result, only a small proportion of tumour cells are exposed to appropriate concentrations of 5-FU at the sensitive phase of the cell cycle. Administration of infusional 5-FU would theoretically increase the exposure of sensitive tumour cells to the drug. Initial studies of protracted intravenous infusion of 5-FU were performed in the treatment of colorectal cancer. These demonstrated that 5-FU 300 mg $m^{-2}$ could be administered by continuous infusion, without interruption, for up to 60 days or up to 36 g cumulative dose (Lockich et al, 1981). Subsequent work showed a higher response rate with infusional than bolus injection (Lokich et al, 1983). Single-agent infusional 5-FU at doses of 200–300 mg $m^{-2}$ daily has been reported to have activity in metastatic breast cancer (Jabboury et al, 1989; Ng et al, 1994), with one study reporting a response rate of 53% in heavily pretreated patients (Huan et al, 1989). In a review of six phase II infusional 5-FU studies in metastatic breast cancer a mean response rate of 29% was reported (Hansen, 1991). Non-randomized studies on large operable breast tumours treated with a combination of infusional 5-FU and bolus epirubicin and cisplatin (ECF) show response rates of up to 98% (Smith et al, 1993, 1995). Furthermore, an overall response rate of 84% has been reported for ECF in 43 patients with metastatic (29 patients) and locally advanced (14 patients) breast cancer including a complete response rate of 24% in the metastatic patients (Jones et al, 1994). Both the unique combination of agents together with the higher overall dose of 5-FU administered in each cycle compared with that normally given in bolus regimens may have contributed to this encouraging anti-tumour activity.

© Cancer Research Campaign 1998

The present study set out to establish the best-tolerated dose of cyclophosphamide and methotrexate, given in a modified 'classical' schedule, that could be used concomitantly with continuous infusional 5-FU at a dose of 200 mg $m^{-2}$ in the treatment of metastatic or relapsed breast cancer. This study demonstrates that cyclophosphamide 480 mg $m^{-2}$, days 1 and 8, and methotrexate 40 mg $m^{-2}$, day 1, is well tolerated when combined with infusional 5-FU in the treatment of metastatic breast cancer.

Fatigue/lethargy was the most frequently reported side-effect, being seen in just over half the patients in our study. Fatigue was severe (grade III) in three patients. One patient with persistent grade II fatigue elected to discontinue her treatment for this reason. Fatigue/lethargy is a well-recognized complication of CMF and our findings are in keeping with those reported for the classical CMF chemotherapy regimen (Smith et al, 1993). The palmar–plantar syndrome, a complication of 5-FU, is seen more frequently with infusional treatment in which up to 26% of patients develop grade III toxicity (Jones et al, 1994; Smith et al, 1995). We observed grade II palmar–plantar syndrome in only one patient. Oral pyridoxine has been reported as an effective therapy when this condition occurs as a side-effect of 5-FU treatment (Fabian et al, 1990). In keeping with this, our patient's symptoms resolved on pyridoxine 50 mg t.i.d. p.o. Patients tolerated the ambulatory pump well as reported in previous studies (Ng et al, 1994).

DVT occurred in 24% of our patients. In four cases this developed in relation to the Hickman line, despite the use of prophylactic warfarin from the day of its insertion. In a study of 322 indwelling venous devices, Eastridge et al (1995) observed a 10% thrombosis rate not related to coagulation profiles of the patients. Infusional 5-FU resulted in superior vena caval thrombosis in 9% of patients in the Edinburgh study (Ng et al, 1994). It is possible that cyclophosphamide exacerbates phlebitis induced by continuous 5-FU infusion, leading to an increased risk of thrombosis.

In a study evaluating a 21-day schedule of infusional CMF in 28 patients, cyclophosphamide 750 mg $m^{-2}$ and methotrexate 50 mg $m^{-2}$ were given as i.v. bolus injections on day 1 only. The infusional 5-FU was administered as in the present study. Despite the fact that 23 patients had received previous chemotherapy, the overall response rate was 50%. This included two patients with a complete response who had had anthracycline-containing regimens for metastatic liver and extensive locally recurrent disease. The 21-day regimen was well tolerated, grade III neutropenia being observed in 12 patients, grade III mucositis in nine and grade II palmar–plantar syndrome in four (Mackay et al, 1996). These results are consistent with the findings of our study, in which 36% response rate (CI = 18–58%) was seen. Although precise details of the DI of cyclophosphamide achieved is not given in their study, the planned DI, 35.7 mg $m^{-2}$, is similar to that of the planned regimen received by patients in cohorts 3 and 4 of our study, 34.3 mg $m^{-2}$.

This study demonstrates that cyclophosphamide 480 mg $m^{-2}$, day 1 and 8, and methotrexate 40 mg $m^{-2}$, day 1, appear well tolerated in combination with continuous infusional 5-FU, 200 mg $m^{-2}$ $day^{-1}$. Taken in conjunction with the results reported by Mackay et al (1996), this study supports the contention that infusional CMF chemotherapy may have a role to play in the management of metastatic breast cancer. These findings have laid the groundwork for a randomized trial comparing infusional CMF with conventional CMF and other combination regimens in the treatment of locally advanced and metastatic breast cancer.

### REFERENCES

Bern MM, Lokich JJ, Wallach SR, Bothe Jr A, Benotti PN, Arkin CF, Greco FA, Huberman M and Moore C (1990) Very low doses of warfarin can prevent thrombosis in central venous catheters. *Ann Intern Med* **112**: 423–428

Bonnadona G and Valagusa P (1985) CMF based adjuvant chemotherapy in resectable breast cancer. *Breast Cancer Res Treat* **5**: 95–115

Brandi M, De-Mitrio A, Ditonno P, Catino A, Lorusso V and DeLena M (1994) Oral versus intravenous CMF in metastatic breast cancer: A randomised study. *Int J Oncol* **4**: 559–565

Brown-Smith JK, Stoner MH and Barley Z (1990) Tunneled catheter thrombosis: factors related to incidence. *Oncol Nursing Forum* **17**: 543–549

Capri G, Tarenzi E, Fulfaro F and Gianni L (1996) The role of taxanes in the treatment of breast cancer. *Semin Oncol* **23** (1 suppl. 2): 68–75

Christophidis N, Vajda FJE, Lucas I, Drummer O, Moon WJ and Louis WJ (1978) Fluorouracil therapy in patients with carcinoma of the large bowel: a pharmacokinetic comparison of various rates and routes of administration. *Clin Pharmacokinet* **3**: 330–336

DeBrujn EA, Driessen OM and Hermans J (1991) The CMF regimen. Toxicity patterns following stepwise combinations of cyclophosphamide, methotrexate and fluorouracil. *Int J Cancer* **48**: 67–72

Eastridge BJ and Lefor AT (1995) Complications of indwelling venous access devices in cancer patients. *J Clin Oncol* **13**: 233–238

Engelsman E, Klijn JCM, Rubens RD, Wildiers J, Beex LVAM, Nooij MA, Rotmensz N and Sylvester R (1991) 'Classical CMF' versus a 3-weekly intravenous CMF schedule in post-menopausal patients with advanced breast cancer. *Eur J Cancer* **27**: 966–970

Fabian CJ, Molina R, Slavik M, Dahlberg S, Giri S and Stephens R (1990) Pyridoxine therapy for palmar–plantar erythrodysesthesia associated with continuous 5-fluorouracil infusion. *Invest New Drugs* **8**: 57–63

Falkson G, Tormey DC, Carey P, Witte R and Falkson HC (1991) Long-term survival of patients treated with combination chemotherapy for metastatic breast cancer. *Eur J Cancer* **27**: 973–977

Greene D, Nail LM, Fieler VK, Dudgeon D and Jones LS (1994) A comparison of patient-reported side-effects among three chemotherapy regimens for breast cancer. *Cancer Pract* **2**: 57–62

Hansen R, Quebbeman E, Beatty P, Ritch P, Anderson T, Jenkins D, Frick J and Ausman R (1987) Continuous 5-fluorouracil infusion in refractory carcinoma of the breast. *Breast Cancer Res Treat* **10**: 145–149

Hansen RM (1991) 5-fluorouracil by protracted venous infusion: a review of recent clinical studies. *Cancer Invest* **9**: 637–642

Hayes DF, Henderson IC and Shapiro C (1995) Treatment of metastatic breast cancer: present and future prospects. *Semin Oncol* **22** (Suppl. 5): 5–21

Heggie GD, Sommadossi JP, Cross DS, Hasler WJ and Diasio RB (1987) Clinical pharmacokinetics of 5-fluorouracil and its metabolites in plasma, urine and bile. *Cancer Res* **47**: 2203–2206

Henderson IC, Hayes DF and Gelman R (1988) Dose–response in the treatment of breast cancer: a critical review. *J Clin Oncol* **6**: 1501–1515

Huan S, Pazdur R, Singhakowinta A, Bohumil S and Vaitkevicius VK (1989) Low dose continuous infusion 5-fluorouracil. Evaluation in advanced breast carcinoma. *Cancer* **63**: 419–422

Jabboury K, Holmes FA and Hortobagyi G (1989) 5-fluorouracil rechallenge by protracted infusion in refractory breast cancer. *Cancer* **64**: 793–797

Jones AL, Smith IE, O'Brien ME, Talbot D, Walsh G, Ramage F, Robertshaw H and Ashley S (1994) Phase II study of continuous infusion fluorouracil with epirubicin and cisplatin in patients with metastatic and locally advanced breast cancer: an active new regimen. *J Clin Oncol* **12**: 1259–1256

Lokich J, Bothe A, Fine N and Perri J (1981) Phase I study of protracted venous infusion of 5-fluorouracil. *Cancer* **48**: 2565–2568

Lokich J, Fine N, Perri J and Bothe A (1983) Protracted ambulatory venous infusion of 5-fluorouracil. *Am J Clin Oncol Cancer Clin Trial* **6**: 103–107

Luck HJ, Thomssen C, Dubois A, Lisboa BW, Untch M, Kuhnle H, Konecny G, Janicke F, Meerpohl HG, Lindner C, Hecker D and Diergarten K (1996) Interim analysis of a phase II study of epirubicin and paclitaxel as first-line therapy in patients with metastatic breast cancer. *Semin Oncol* **23** (1 suppl. 1): 33–36

Mackay J, Cameron DA, Gardiner J, Leonard T, Lee LE, Leonard RCF (1996) A pilot study of infusional CMF (CMF-inf): Active and well tolerated in breast cancer. *Ann Oncol* **7**: 409–411

McDermott BJ, Vanderbers HW and Murphy RF (1982) Non-linear pharmacokinetics for the elimination of 5-fluorouracil after intravenous administration in cancer patients. *Cancer Chemother Pharmacol* **9**: 173–179

© Cancer Research Campaign 1998

Ng JSY, Cameron DA, Lee L, Dixon JM and Leonard RCF (1994) Infusional 5-fluorouracil given as a single agent in relapsed breast cancer: its activity and toxicity. *Breast* **3**: 87–89

Pfeiffer P, Cold S and Rose C (1992) Cytotoxic treatment of metastatic breast cancer. Which drugs and drug combinations to use? *Acta Oncol* **31**: 219–224

Shapiro CL, Gelman RS, Hayes DF, Osteen R, Obando A, Canellos GP, Frei III, E and Henderson IC (1993) Comparison of adjuvant chemotherapy with methotrexate and fluorouracil with and without cyclophosphamide in breast cancer patients with one to three positive axillary lymph nodes. *J Natl Cancer Inst* **85**: 812–817

Smith IE and Powles TJ (1993) MMM (mitomycin/mitoxantrone/methotrexate): an effective new regimen in the management of metastatic breast cancer. *Oncology* **50** (Suppl. 1): 9–15

Smith IE, Jones AL, O'Brien ME, McKinna JA, Sacks N and Baum M (1993) Primary medical (neo-adjuvant) chemotherapy for operable breast cancer. *Eur J Cancer* **29**: 1796–1799

Smith IE, Walsh G, Jones AL, Prendiville J, Johnston S, Gusterson B, Ramage F, Robertshaw H, Sacks N, Ebbs S, McKinna JA and Baum M (1995) High complete remission rates with primary neo-adjuvant infusional chemotherapy for large early breast cancer. *J Clin Oncol* **13**: 424–429

Sotos GA, Grogan L and Allegra CJ (1994) Preclinical and clinical aspects of biomodulation of 5-fluorouracil. *Cancer Treat Rev* **20**: 11–49

Spicer DV, Ardalan B, Daniels JR, Silbermann H and Johnson K (1988) Re-evaluation of the maximum tolerated dose of continuous venous infusion of 5-fluorouracil with pharmacokinetics. *Cancer Res* **48**: 459–461

Tannock JF, Boyd NF, DeBoer G, Erlichman C, Fine S, Larocque G, Mayers C, Perrault D and Sutherland H (1988) A randomised trial of two dose levels of CMF chemotherapy for patients with metastatic breast cancer. *J Clin Oncol* **6**: 1377–1387

*© Cancer Research Campaign 1998*